# Determinants of Adherence with Malaria Chemoprophylactic Drugs Used in a Traveler's Health Clinic

**DOI:** 10.1155/2015/163716

**Published:** 2015-08-24

**Authors:** Ibrahim Shady

**Affiliations:** Community Medicine Department, Faculty of Medicine, Mansoura University, Mansoura 35516, Egypt

## Abstract

*Background*. The WHO recommends mefloquine, atovaquone/proguanil, and doxycycline for malaria chemoprophylaxis. Adherence to a drug is determined by many factors. *Objective*. To detect the determinants of travelers' adherence to malaria chemoprophylaxis. *Methods*. A prospective comparative study was conducted from January 2012 to July 2013 that included travelers (928 travelers) to malaria endemic countries who visited the THC. They were classified into 3 groups: the 1st is the mefloquine group (396 travelers), the 2nd is the doxycycline group (370 travelers), and finally those who did not receive any drugs (162 travelers). The participants from the 1st and 2nd groups enrolled in the study. *Results*. Univariate and multivariate analyses were performed. The predictors for adherence in the mefloquine group were travel to an African destination [OR = 51 (6.8–2385)], higher than a secondary school education [OR = 21 (4.1–144.2)], organized travel [OR = 4 (2.1–6.5)], traveling for leisure [OR = 2.1 (1.1–0.4)], and nationality [OR = 2 (1.11–4.00)]. In the doxycycline group, the predictors included higher than a secondary education [OR = 20.1 (4.5–125.1)], organized travel [OR = 11.4 (5.5–20.9)], travel for leisure [OR = 7 (2.3–22.9)], travel to an African destination [OR = 6.1 (0.41–417)], and nationality [OR = 4.5 (2.3–9.5)]. *Conclusion*. Adherence with malaria chemoprophylaxis could be affected by many factors such as nationality, education, and organized travel.

## 1. Background

Malaria is an important threat to tourists, employees, and international travelers traveling or working in endemic areas due to the potentially rapid onset of infection and the severity of the disease. International travelers should be protected from malaria by chemoprophylaxis and prophylactic measures against mosquito bites. Chemoprophylaxis is a key component of malaria prevention because none of the vector protection measures completely protect against mosquito bites during night time activities. However, the effectiveness of chemoprophylaxis is limited by lack of compliance with drug intake [1–3].

Malaria chemoprophylaxis has been shown to have a low cost-benefit ratio compared to other prophylactic interventions for travelers [4], and several studies have suggested that increased risks of malaria may occur in noncompliant travelers [5–8]. Among travelers to endemic areas, compliance regarding malaria chemoprophylaxis is generally poor, ranging from 32 to 74% depending on the definitions used [9, 10].

Risk factors that predict noncompliance are important, as they may be used to increase compliance by improving pretravel information. Such risk factors have been studied among European and North American travelers on return flights from Kenya and several West African countries and have included young age, longer travel duration, previous travel to tropical destinations, visits to friends or family as the travel purpose, occurrence of adverse reactions, and use of the drugs [11, 12].

Kuwait is considered a high income country, and its inhabitants commonly travel during the summer period due to hot weather, as the temperature may reach as high as 50°C. Malaria is not endemic in Kuwait but, with a large expatriate population, the number of imported infections increased every year compared with the increased number of recruiting expatriates. However, malaria infection risk is still very low due to the absence of the vector for malaria transmission [13, 14].

Only one traveler's health clinic is available in Kuwait to provide travel health services, which include immunizations, chemoprophylaxis for malaria, and health advice/information about the prevalent diseases/health hazards in the destination country [15].

Two drugs are used for malaria chemoprophylaxis. These drugs include mefloquine hydrochloride (250 mg tab) (generic: mefloquine or lariam), which is given on weekly basis (i.e., every week starting one week before travel and completed at least 4 weeks after leaving the malaria endemic area), and doxycycline (100 mg caps) (generic: vibramycin or doxydar), which is given on daily basis (i.e., one capsule every day starting one day before travel and completed 4 weeks after leaving the malaria endemic area) [16–20].

Many studies have been conducted to identify factors that influence compliance with medications, most of which have focused on treatments to cure, the ability to reduce or delay complications and symptoms caused by chronic diseases. Only a few studies have assessed compliance in the context of chemoprophylaxis. Recently, the concept of adherence has supplanted compliance, as adherence implies that the patient agrees with the prescribed recommendations rather than passively obeying them [11].

The main objectives of this study are to explore the determinants and risk factors affecting the adherence of travelers visiting the THC to malaria chemoprophylactic drugs.

## 2. Subjects and Methods

Our study is a prospective comparative (analytical) study that included all travelers to malaria endemic areas who visited the THC (928 travelers) to obtain malaria prophylaxis. The study period was from January 2012 to July 2013.

We interviewed 928 travelers who visited the THC to receive their malaria chemoprophylaxis. They consisted of Kuwaitis and residents from other nationalities, mainly Egyptians and Indians, to lesser extent Arabians other than Egyptians, Europeans, Americans, and Asians, and rarely those from South and West African countries. This was consistent with the population demography in Kuwait [21, 22].

All of the 928 travelers were classified on the basis of malaria chemoprophylactic drugs into 3 groups: the 1st group consisted of travelers who received mefloquine (396 travelers, 42.7%), the 2nd group consisted of travelers who received doxycycline (370 travelers, 39.9%), and the last group consisted of travelers who refused to receive malaria drugs or had contraindications to the drugs (162 travelers, 17.5%). The choice of drug was based on many factors, including the traveler's health status, past history of taking the drug without problems, contraindications, and the traveler's decision as reported by Senn et al., 2007, who reported many causes behind travelers' choice of malaria chemoprophylactic drug and the factors that influenced their final decision [19].

Malaria chemoprophylactic drugs were given to all travelers free of charge according to the rules set by the managing department.

All travelers enrolled on this study were interviewed by the same travel health team (physician and/or nurse) and asked to complete a predeparture health questionnaire [20] that included sociodemographic data that consisted of contact information (mobiles, landlines, and emails), travel journey data, and malaria prophylactic drugs.

Fortunately, Kuwait has well-developed communication infrastructure which is cheap and widespread. This communication system enables nearly all of its inhabitants to have more than one mean of communications (emails, mobiles, and landlines). The government holds the control over the landline system which is provided with accepted annual fees for the subscribers. In addition, there are three large mobile providing companies that provide mobile lines in reasonable fees covering all over the country. Also, Internet access is available and it is widely distributed.

We used mobile lines as the first line of communication, followed by landlines and then the Internet. Luckily, most of travelers responded to the mobile contact which is used by some researchers for monitoring the malaria and its treatment [23].

We contacted all of the 766 travelers (there were no defaulters or drop-out cases) who obtained malaria chemoprophylaxis twice after their return (after the 1st week and at the end of the 4th week after their return). They were asked about the regularity with which they took the drug during and after their return. Only those who answered “no” were subjected to another set of questions to determine the factors related to their nonadherence.

Adherence to the chemoprophylaxis regimen was recorded retrospectively according to self-reported use and defined as regular and uninterrupted use of both drugs until the posttravel communication with the travelers [18].

The operational definition of regular and uninterrupted use was not having missed more than one day dose per week on average for the daily dose drug and not having missed any dose of the weekly dose drug. However, the term adherence has recently supplanted compliance, as adherence implies that the patient agrees with the prescribed recommendations rather than passively obeying them [11].

We designed two versions of the questionnaire (Arabic and English). Individuals below 6 years of age were helped by their parents or older brothers/sisters, and individuals who could not understand or complete the questionnaire were assisted in its completion.

Informed consent was obtained from every individual in this study. We obtained approval to conduct our study from the ethical and approval committee of Traveler's Health Board and the head of the department.

The terms adherence and compliance were used interchangeably in this study as compliance constitutes an element of adherence.

### 2.1. Date Collection and Analyses

Data were collected and analyzed using SPSS for Windows version 17.0 (SPSS, Chicago, IL). Continuous data are expressed as the mean ± SD. Comparisons of the continuous data between the two groups were performed using independent Student's *t*-tests. All categorical data are expressed in number and percent and were compared between the two groups using Pearson's and Fisher's exact *χ* ^2^ tests. The 95% confidence intervals (CI) for the differences in means were calculated. Statistical significance was set at *p* < 0.05. Multivariate analysis was performed to determine the factors that affect adherence to the drug regimens, and the variables of the multivariate analysis were selected from the most significant variables in the univariate analysis.

## 3. Results and Comments

The sociodemographic and travel-related data of the 928 subjects enrolled initially in this study were obtained and are described in Table 1, which shows that males represent slightly less than 2/3 of individuals in the groups that received mefloquine (group 1) and doxycycline (group 2), but this proportion was inverted for the no chemoprophylaxis group (group 3). Nonnationals represented approximately 60% of the 3 groups. Most travelers in groups 1 and 2 were educated above the secondary level; however, for the 3rd group, postgraduate education was higher. Most of the travelers in the 3 groups were working (80.8% to 87.7%). Many people were travelling for leisure purposes (59.6% to 70.4%) followed by work purposes and then for family visits. Organized trips were more prevalent than independent trips. African countries were the destination of choice for groups 1, 2, and 3 (73.7%, 78.4%, and 59.3%, resp.), followed by Asian countries and Latin countries. The mean age of the travelers was between 32 and 38.5 years, and the mean travel duration was 3.5 to 4.1 weeks.

### 3.1. Use of Chemoprophylaxis and Adherence

**Figure 1:**
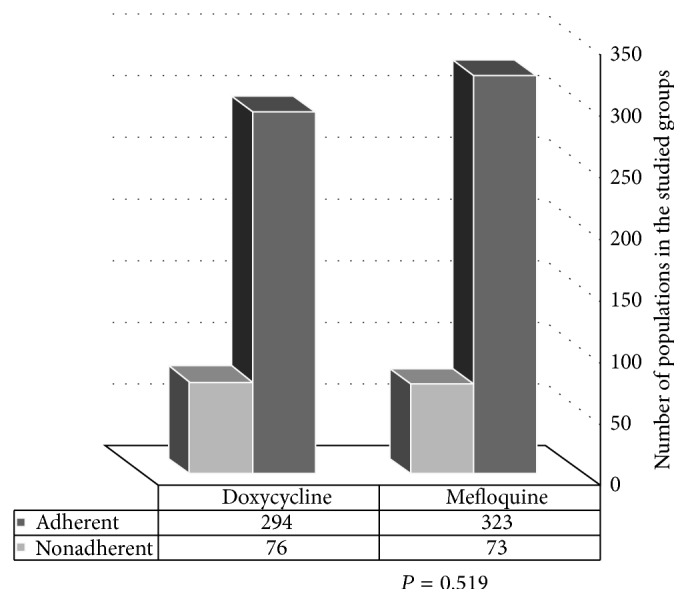
Comparison between the mefloquine and doxycycline groups according to adherence.

Of the 928 subjects initially enrolled on the study, only 82.5% obtained the chemoprophylactic drugs against malaria, and these individuals were classified into 2 groups; the 1st group was the mefloquine group, and the 2nd group was the doxycycline group. The rates of nonadherence for groups 1 and 2 were 18.4% and 20.5%, respectively [Figure 1]. In the 1st group, most of the nonadherence occurred after finishing travel (27.4% dropped out after the 1st week and 28.8% dropped out after the 4th week) as opposed to the 2nd group (13.2% dropped out after the 1st week and 14.5% dropped out after the 4th week), and the differences were statistically significant (*p* = 0.03). However, a larger proportion of the second group dropped out during travel (32.9%) in contrast to the 1st group (6.8%), and the difference was highly statistically significant (*p* = 0.0001).

### 3.2. Factors Affecting Adherence to Malaria Chemoprophylaxis

Tables 3 and 4 show the univariate analysis of adherence for the mefloquine and doxycycline groups with the risk factors of interest. Some risk factors were significantly associated with nonadherence in both groups. Nationality significantly affected the degree of adherence, specifically by 2.25-fold in group 1 and 5.1-fold in group 2. Regarding education, after using noneducated individuals as a reference value for comparison, we found that the level of education maximally affected the degree of adherence for individuals with higher than a secondary level of education (24.6- and 22.3-fold for group 1 and group 2, resp.), and the differences were statistically significant (*p* = 0.0001). However, nonadherence was maximal among university educated individuals in groups 1 and 2 (27.4% and 30.3%, resp.), and the differences were statistically significant (*p* = 0.005). Regarding occupation, after using the nonworking group as a reference value for comparison, we found that nonadherence was increased among blue collar workers for groups 1 and 2 (35.6% and 25%, resp.) and the differences were statistically significant (*p* = 0.0001). Additionally, the domestic helper occupation category showed significant nonadherence tendencies in both groups. Traveling for leisure was a very important factor in determining adherence in groups 1 and 2 (71.8% and 67.7%, resp., *p* = 0.0001). Additionally, travel that was organized by an agent affected the tendency for adherence more than travel that was arranged independently, and the difference was statistically significant for both groups (*p* = 0.0001). Travel destination to African countries was a significant factor for adherence and compliance to the drug and its dosing for the complete regimen and produced 53-fold higher adherence for the mefloquine group (group 1) and 8.6-fold higher adherence for the doxycycline group (group 2). The travel duration was also a significant factor for adherence to the given drug (*p* = 0.0001).

Table 5 shows the multivariate analysis for selected risk factors that predicted good adherence for both groups. We found that predictors of good adherence for the mefloquine group included travel to an African destination, education above a secondary level, organized travel, traveling for leisure, and Kuwaiti nationality.

However, in the doxycycline group, the predictors included higher than a secondary level of education, followed by organized travel, and then travel for leisure, travel to an African destination, and Kuwaiti nationality.

## 4. Discussion

This study identified both collective and individual determinants of correct adherence to malaria chemoprophylaxis among individuals travelling to malaria endemic areas and visiting the THC during the period from January 2012 to July 2013.

### 4.1. Use of Chemoprophylaxis and Adherence

Of the 928 subjects initially enrolled on the study, only 82.5% received chemoprophylactic drugs against malaria due to many factors such as refusing to take the antimalarial drugs due to bad previous experience and/or contraindications to the drugs [19, 24, 25]. Individuals who received the drugs were classified into 2 groups; the 1st group was the mefloquine group, and the 2nd group was the doxycycline group (Table 2).

The rate of adherence for groups 1 and 2 was 81.6% and 79.5%, respectively, as shown in Figure 1. This rate is higher than that reported in the study by Frank G. J. and Anne L. K. (1991-1992), which examined adherence to malaria chemoprophylaxis among Dutch travelers [26] and reported an overall adherence of 59%. This difference may be due to differences in the types of subjects enrolled in the study, as we enrolled all individuals who visited the THC for travel health advice and measures; however, the Dutch study was a part of larger cohort study of health risks among travelers. In addition, our adherence results were higher than those reported by other studies [26–33]. In our study the rates of nonadherence to both drugs were nearly the same which might be due to travelers' beliefs that they had a lower risk of malaria infection on returning to Kuwait due to the fact that malaria is not endemic in Kuwait [14]. Other causes that lead to nonadherence included simply forgetting, fear of long term side effects, discontinuation due to occurrence of unpleasant side effects [such as neuropsychiatric (nightmares, vivid dreams, anxiety, insomnia, and depression), gastrointestinal (heart burns, colic, nausea, and vomiting), and musculoskeletal (easy fatigability, muscle pains, and lassitude)], and busy life style.

In the 1st group, most of the nonadherence occurred after finishing travel (27.4% dropped out after the 1st week and 28.8% dropped out after the 4th week) in contrast to the 2nd group (13.2% dropped out after the 1st week and 14.5% dropped out after the 4th week), and the differences were statistically significant (*p* = 0.03). However, for the second group, more individuals dropped out during travel (32.9%) than in the 1st group (6.8%), and the difference was highly statistically significant (*p* = 0.0001). Our results regarding the mode of nonadherence are much lower than those reported by the Dutch study [26], which indicated that half of all noncompliance was due to early discontinuation after return. More than half of the travelers had stopped their chemoprophylaxis because they found that it was unnecessary to continue the treatments. However, in our study, the lower rate of nonadherence as well as the lower rate of discontinuation, either early or late after return, may be attributed to the travel-related information provided to the travelers during their visits to the THC. Nonadherent individuals reported that they found it inconvenient to continue to take the medications for 4 weeks after return due to their busy schedules. Others believed that they were not exposed to malaria during their travels; however, some individuals experienced unpleasant side effects, as reported in some studies [24, 25].

### 4.2. Factors Affecting Adherence to Malaria Chemoprophylaxis

A univariate analysis was conducted to determine which factors affect the traveler's adherence to the chemoprophylactic drugs (mefloquine group, Table 3, and doxycycline group, Table 4).

Tables 3 and 4 show the factors of both groups of travelers. Nationality increases the degree of adherence in both groups (by 2.25-fold in the mefloquine group and 5.1-fold in the doxycycline group) which could be attributed to the fact that some non-Kuwaiti individuals usually travel during the holidays to their home countries and they believe that they are not at risk for acquiring malaria. The nationality of the travelers affected the adherence rate through factors such as the belief that malaria is not endemic in the country due to the absence of the vector transmitting it. So, Kuwaitis tended to be nonadherent especially when they return to their home country. Non-Kuwaitis who come from areas where malaria is endemic know the impact of malaria on their health as well as the long duration of treatment so their adherence rate is higher. Non-Kuwaitis from developed countries have a high amount of health information about travel-related health hazards leading them to be more adherent.

Education significantly affected the degree of adherence for both groups, and the maximum difference was observed in individuals with higher than a secondary level of education (24.6-fold and 22.3-fold); however, a university level of education significantly increased the degree of nonadherence in both groups. This could be because those educated above secondary level usually adhere to advice to take a drug due to their psychological condition regarding their health. Also, those educated above secondary level showed an increased level of awareness of antimalarial chemoprophylaxis, which served to motivate them to adhere to the prescribed drug regimens. However, individuals with a university level of education had enormous information regarding the destination, drug, and disease, which helped them to decide whether to follow or not follow our advice. In contrast to our results, Gagneux et al., 1996 [34], found no association between the adherence and the degree of education. Employment status also affected the degree of adherence in both groups, as adherence was insignificantly high among white collar workers in both groups. However, nonadherence was significantly higher among blue collar workers in both groups, which could be because white collar workers are usually highly educated (at least above the secondary level). However, blue collar workers usually do not complete their education level to a secondary level. Degree of adherence among domestic helpers usually is affected by the degree of adherence of their sponsors as they usually accompany their sponsors during their vacations so they usually follow their mode of adherence as well as the type of prophylaxis they got.

Travel for the purpose of leisure significantly affected the degree of adherence in both groups, which could be attributed to the fact that leisure travelers tended to go on safari and other travel-related adventures such as visiting wild life fields and forests and climbing mountains. They believed that these adventures are risky because they occur in open places; therefore, they likely tend to be more compliant than others. In organized travel, where travelers are on an organized trip, they are more likely to be adherent. Similar results were identified in the Dutch study in 1997 [26]. This could be explained because group travelers (organized tours) usually stimulate and encourage all participants to be compliant by social stimulation [26]. Another explanation is that organized tours are usually conducted by highly experienced tourist companies, and they obtain all of the health education materials that contain the risks of the travel destination and how they could be prevented. These companies disseminate this information to their travelers. Travel destinations in Africa significantly promoted adherence in both groups because most travelers to these areas are aware that malaria in African countries is highly endemic and is evenly distributed; therefore, the malaria risk for travelers is the highest in the world [9, 12], which encouraged the high adherence reported in this study. Additionally, travelers to African countries are usually exposed to travel-related hazards as they go on safari, visit open zoos, climb mountains, and live in camps and tents. They believe that such activities increase their risk of getting malaria because they occur in open places. To a lesser extent, travelers believe that the degree of health care in these countries is not high and this makes them more adherent. Also, most trips to African destinations were organized by travel agencies that ask every traveler to provide proof of malaria chemoprophylaxis as a prerequisite for getting an entrance visa. Similar results were reported by other studies [11, 20]. Age > 40 years and travel duration ≥ 5 weeks encouraged travelers to be more compliant, and similar results were reported in other studies [11, 12, 26].

Predictors of good adherence for both groups included five factors that differed in the order and strength for both groups. However, our results are very similar to those of the Dutch study [26]. This study provided valuable information for designing plans to increase adherence for those with a lower adherence rate and to strengthen adherence among those with higher rates of adherence.

## 5. Conclusion and Recommendations

Adherence with malaria chemoprophylaxis could be affected by many risk factors such as education, organized travel, leisure travel, and travel destination, as well as the duration of travel and the age of traveler. Therefore, adherence with malaria chemoprophylaxis should be improved in travelers to areas with high risks of malaria infection by designing health education programs that must be provided to those travelers in many occasions and periodically.

Travelers' health education programs should be designed with special emphasis on the need for a clear conveyance of the importance of continuing chemoprophylaxis.

Additionally, new methods of traveler education need to be developed and should be based on data from in-depth studies of travelers' motivations to take preventative measures.

Extra attention should be given to young, independent travelers and to those who have been found to show a low rate of adherence and may underestimate their risk of malaria on the basis of their thoughts and beliefs.

## Supplementary Material

This is a modified pre-departure health questionnaire that was modified from the pre-departure health questionnaire provided by WHO in the International Travel and Health.
It was modified to be suitable to our aim of the study which is the assessment of the adherence to the malaria chemo-prophylactic drugs given in the THC.
We added the drug related data and the following part to suit our aim.
We removed some detailed travel related data from the original pre-departure heath questionnaire provided by the WHO as it is not necessary in our study.

## Figures and Tables

**Table 1:** Sociodemographic characteristics of the studied groups.

Characteristic	Mefloquine group		Doxycycline group		No chemoprophylaxis	
	*N*	(%)	*N*	(%)	*N*	(%)
Total = 928 (100)	**396**	**100**	**370**	**100**	**162**	**100**
Sex						
Male	247	62.4	231	62.4	71	43.8
Female	149	37.6	139	37.6	91	56.2
Nationality						
Non-Kuwaiti	241	60.9	222	60.0	101	62.3
Kuwaiti	155	39.1	148	40.0	61	37.7
Education						
Not educated	11	2.8	15	4.1	5	3.1
Primary, secondary	89	22.5	78	21.1	32	19.8
Above secondary	159	40.2	122	33.0	35	21.6
University	73	18.4	59	16.0	12	7.4
Postgraduate	64	16.2	96	26.0	81	50
Occupation						
Not working	73	18.4	71	19.2	20	12.3
White collar	189	47.7	2.3	54.9	88	54.3
Blue collar	57	14.4	33	8.9	21	13
Domestic helper	77	19.4	63	17	33	20.4
Travel purpose						
Leisure	236	59.6	242	65.4	114	70.4
Family visit	52	13.1	28	7.6	12	7.4
Work	108	27.3	100	27	36	22.2
Travel style						
Independent	120	30.3	96	25.9	36	22.2
Organized	276	69.7	274	74.1	126	77.8
Destination						
Africa	292	73.7	290	78.4	96	59.3
Asia	96	24.2	77	20.8	24	14.8
South America	8	2.1	3	0.8	42	25.9

	Mean	SD	Mean	SD	Mean	SD

Age (years)	38.5	11.9	37.5	12.4	32.6	13.2
Travel duration (W)	4.0	1.0	4.1	1.2	3.5	1.1

**Table 2:** Nonadherence types among the studied groups.

Types of nonadherence	Mefloquine group	Doxycycline group	*P* value	OR ± 95 CI
Not started	12 (16.4)	5 (6.6)	0.05	
Irregular use	15 (20.5)	25 (32.9)	0.08	052 ± (0.25–1.11)
Dropped during travel	5 (6.8)	25 (32.9)	0.0001	0.5 ± (0.05–0.4)
Dropped after 1st week of return	20 (27.4)	10 (13.2)	0.03	2.5 ± (1.1–2.9)
Dropped after 4th week of return	21 (28.8)	11 (14.5)	0.03	2.4 ± (1.1–5.5)
Total	**73 (100)**	**76 (100)**		**149 (100)**

**Table 3:** Univariate analysis of mefloquine group between adherent and nonadherent individuals.

Characteristic	Nonadherent		Adherent		*P* value	OR ± 95 CI
	*N *	(%)	*N *	(%)		
Total = 149 (100)	**73**	**100**	**323**	**100**		
Sex						
Male	45	61.7	202	62.5	0.4	0.96 (0.57–1.63)
Female	28	38.3	121	37.5		
Nationality						
Non-Kuwaiti	55	75.3	186	57.6	0.002	2.25 (1.28–4.08)
Kuwaiti	18	24.7	137	42.4		
Education						
Not educated^*∗*^	8	11	3	0.9		
Primary, secondary	17	23.3	72	22.3	0.0005	10.9 (2.32–70.6)^#^
Above secondary	15	20.5	144	44.6	0.0001	24.6 (5.23–159.3)^#^
University	20	27.4	53	16.4	0.005	6.87 (1.46–44.3)^#^
Postgraduate	13	17.8	51	15.8	0.001	10.3 (2.43–45.04)^#^
Occupation						
Not working^*∗*^	6	8	67	20.7		
White collar	25	34.2	164	50.8	0.13	0.6 (0.21–1.44)
Blue collar	26	35.6	31	9.5	0.0001	0.11 (0.04–0.28)
Domestic helper	16	21.9	61	18.9	0.01	0.34 (0.12–0.92)
Travel purpose						
Family visit^*∗*^	18	24.7	34	10.5		
Leisure	34	46.6	232	71.8	0.0001	3.6 (1.8–7.1)
Work	21	28.8	87	26.9	0.01	2.18 (1.03–4.63)
Travel style						
Independent	43	58.9	77	23.8	0.0001	4.6 (2.7–7.8)
Organized	30	41.1	246	76.2		
Destination						
South America^*∗*^	7	9.5	1	0.3		
Asia	33	45.2	63	19.5	0.004	13.05 (1.57–611.5)^#^
Africa	33	45.2	259	80.2	0.0001	53.6 (7.96–2485)^#^

	Mean	SD	Mean	SD	*t*-test	

Age (years)	38.5	11.9	42.6	10.3	0.003	
Travel duration (W)	4.0	1.0	5.2	1.1	0.0001	

^*∗*^Reference value for comparison.

^#^Fisher's exact test (chi square).

**Table 4:** Univariate analysis of doxycycline group between adherent and nonadherent individuals.

Characteristic	Nonadherent		Adherent		*P* value	OR ± 95 CI
	*N*	(%)	*N*	(%)		
Total = 396 (100)	**76**	**100**	**294**	**100**		
Sex						
Male	53	69.7	178	60.5	0.07	1.5 (0.87–1.61)
female	23	30.1	116	39.5		
Nationality						
Non-Kuwaiti	65	85.5	157	53.4	0.0001	5.1 (2.66–10.58)
Kuwaiti	11	14.5	137	46.6		
Education						
Not educated^*∗*^	12	15.8	3	1.0		
Primary, secondary	16	21.1	62	21.1	0.0001	14.9 (3.5–92.1)^#^
Above secondary	18	23.7	104	35.4	0.0001	22.3 (5.33–135.1)^#^
University	23	30.3	36	12.2	0.004	6.1 (1.4–37.4)^#^
Postgraduate	7	9.2	89	30.3	0.0001	46.9 (9.9–320.7)^#^
Occupation						
Not working^*∗*^	10	13.2	61	20.7		
White collar	20	26.3	183	63.3	0.2	1.5 (0.63–3.35)
Blue collar	19	25	14	4.8	0.0001	0.12 (0.05–0.32)
Domestic helper	27	35.5	36	12.2	0.0001	0.22 (0.09–0.5)
Travel purpose						
Family visit^*∗*^	21	27.6	7	2.4		
Leisure	43	56.6	199	67.7	0.0001	13.7 (5.6–36.6)
Work	12	15.8	88	29.9	0.0001	21.1 (7.6–64.3)
Travel style						
Independent	52	68.4	44	15.0	0.00001	12.2 (6.9–22.1)
Organized	24	31.6	250	85.0		
Destination						
South America^*∗*^	2	2.6	1	0.3		
Asia	20	26.3	57	19.4	0.06	5.6 (0.27–343)^#^
Africa	54	71.1	236	80.3	0.09	8.6 (0.44–517.1)^#^

	Mean	SD	Mean	SD	*t*-test	

Age (years)	37.5	12.4	40.6	9.6	0.02	
Travel duration (W)	4.1	1.2	5.0	1.2	0.0001	

^*∗*^Reference value for comparison.

^#^Fisher's exact test (chi square).

**Table 5:** Multivariate analysis of some risk factors in both groups.

Variables	Group			
	Mefloquine group		Doxycycline group	
	Adj. OR	*P*	Adj. OR	*P*
Kuwaiti nationality	2 (1.11–4.0)	<0.05	4.5 (2.3–9.5)	<0.001
Above secondary	21 (4.1–144.2)	<0.001	20.1 (4.5–125.1)	<0.001
White collars	0.11 (0.04–0.33)	>0.05	1 (0.5–2.1)	>0.05
Leisure travel	2.1 (1.1–3.4)	<0.05	7 (2.3–22.9)	<0.05
Organized travel	4 (2.1–6.5)	<0.05	11.4 (5.5–20.9)	<0.001
African destination	51 (6.8–2385)	<0.001	6.1 (0.41–417)	=0.05
