# Supplementary material for: Determinants of Adherence with Malaria Chemoprophylactic Drugs Used in a Traveler's Health Clinic
Source: J Trop Med. 2015 Aug 24;2015:163716. doi: 10.1155/2015/163716 (PMC4561335; doi:10.1155/2015/163716)
Supplement: Supplementary file 1 — This is a modified pre-departure health questionnaire that was modified from the pre-departure health questionnaire provided by WHO in the International Travel and Health. It was modified to be suitable to our aim of the study which is the assessment of the adherence to the malaria chemo-prophylactic drugs given in the THC. We added the drug related data and the following part to suit our aim. We removed some detailed travel related data from the original pre-departure heath questionnaire provided by the WHO as it is not necessary in our study. [file 163716.f1.docx]

**(No., …)**

**Modified Pre-departure Health Questionnaire**

## Socioeconomic Data:

Name (Optional):

Gender: Nationality:

DOB: Occupation:

Residence: Education:

Mobile : Landline Telephone:

Email (s):

## Travel Related Data:

Travel Destination ……...

Purpose of Travel ………

Travel Style ………

Duration of Stay ………

## Drug related Data:

1. Mefloquine: (Yes No)
2. Doxycycline: (Yes No)
3. No Drug taken (Yes No)

If yes, why?

**Asked after return:**

**Contact after 1^st^ week:**

Adherent (Yes No)

*If Not,*

*Causes of non-adherence:*

*Types of non-adherence:*

Not started (Yes No)

Irregular use (Yes No)

Dropped dose. (Yes No)

If yes, when?

**Contact after 4^th^ week:**

Adherent (Yes No)

*If Not,*

*Causes of non-adherence:*

*Types of non-adherence:*

Dropped dose. (Yes No)

If yes, when?
